# A randomized prospective clinical study evaluating the effectiveness of the Beautibond Xtreme adhesive system using different bonding techniques in class I and II restorations: one-year results

**DOI:** 10.1590/1678-7757-2025-0228

**Published:** 2025-09-16

**Authors:** Walter RAUCCI, Ana Flávia Simões BARBOSA, Adrielle Fracaroli BALTAZAR, Larissa Fernanda PEREIRA, Yara Teresinha Corrêa SILVA SOUSA, Danielle Cristine Furtado MESSIAS, Taylane Soffener Berlanga de ARAUJO

**Affiliations:** 1 Universidade de São Paulo Faculdade de Odontologia de Ribeirão Preto Departamento de Odontologia Restauradora Ribeirão Preto SP Brasil Universidade de São Paulo, Faculdade de Odontologia de Ribeirão Preto, Departamento de Odontologia Restauradora, Ribeirão Preto, SP, Brasil.; 2 Universidade de Ribeirão Preto Programa de Odontologia Ribeirão Preto SP Brasil Universidade de Ribeirão Preto (UNAERP), Programa de Odontologia, Ribeirão Preto, SP, Brasil.; 3 Indiana University Department of Comprehensive Care & Allied Profissions Indiana USA Indiana University, Department of Comprehensive Care & Allied Profissions, Indiana, USA.

**Keywords:** Composite resins, Dental adhesives, Clinical trial, Adhesive techniques, Randomized controlled trial

## Abstract

**Objective:**

This randomized, parallel-group clinical trial aimed to evaluate the one-year clinical performance of the Beautibond Xtreme adhesive system (Shofu Inc., Kyoto, Japan) applied with different bonding strategies in Class I and Class II posterior restorations.

**Methodology:**

A total of 22 patients (14 female and 8 male, aged ≥18 years) requiring restorative treatment provided 152 teeth with Class I or Class II carious lesions or defective restorations. Restorations were randomly assigned to six groups, including two control groups (Class I and II using the total-etch technique) and four test groups (Class I and II using either the self-etch technique or selective enamel etching). All restorations were performed using Beautibond Xtreme adhesive combined with Beautifill LS composite resin. Clinical performance was assessed at baseline, six months, and one year using modified United States Public Health Service (USPHS) criteria, including anatomic form, marginal adaptation, marginal discoloration, color match, surface texture, secondary caries, postoperative sensitivity, and retention. Randomization was performed with a computer-generated sequence, and two calibrated, blinded examiners (Kappa = 0.84) conducted all evaluations.

**Results:**

No significant changes were observed in anatomic form, color match, surface texture, secondary caries, postoperative sensitivity, or retention over time in any group (p>0.05). However, restorations performed using the self-etch technique showed significant deterioration in marginal adaptation and marginal discoloration from baseline to six months and one year (p<0.05). Significant differences were also observed when comparing these restorations to the total-etch and selective enamel etching groups (p<0.05).

**Conclusion:**

The Beautibond Xtreme adhesive system demonstrated better clinical performance when applied with total-etch or selective enamel etching techniques compared to the self-etch mode for both Class I and Class II restorations after one year of follow-up.

## Introduction

Restorative dentistry has evolved significantly with the introduction of adhesive systems, enabling a strong and durable bond between restorative materials and dental tissues. The formation of a hybrid layer, where polymerized monomers infiltrate demineralized dentin, is critical for achieving long-lasting adhesive restorations.^1^ Over time, the development of universal adhesives has provided clinicians with greater versatility, enabling application in etch-and-rinse, self-etch, or selective enamel etching modes, optimizing adhesion while reducing chair time and operator sensitivity.^2^

A major concern in adhesive dentistry is the long-term stability of the adhesive interface, particularly in dentin bonding. The activation of matrix metalloproteinases (MMPs) in acidic environments, hydrolysis, and polymer instability can lead to degradation of the adhesive layer over time. In this context, self-etch adhesives have been proposed as a solution to minimize collagen degradation, as they partially retain the smear layer, limiting dentin demineralization and promoting chemical interaction between acidic monomers and hydroxyapatite.^3,4^

However, despite simplifying the technique, one-step self-etch systems are chemically unstable due to an imbalance in their adhesive mixtures. This instability leads, over time, to phase separation of their components, resulting in a porous and poorly polymerized layer. Moreover, the high content of hydrophilic monomers compromises one-step systems, as they trap water in the adhesive layer and accelerate interface degradation.^3^

Considering the great variability of materials and techniques available for direct restorations, the development of clinical studies is of great importance, as materials are observed in the real-world environment, incorporating all variables of the clinical routine. This allows the construction of evidence-based practice. Therefore, randomized clinical trials provide significant evidence that can contribute to clinicians’ decision-making, considering the different compositions of adhesive systems and whether new usage protocols could provide greater restorative durability.^5,6^

A promising innovation in adhesive technology is Giomer-based restorative materials, which incorporate Surface Pre-Reacted Glass (S-PRG) fillers. These materials exhibit bioactive properties, releasing and recharging fluoride, inhibiting bacterial activity, and promoting remineralization of the surrounding tooth structure.^7^ Clinical studies have demonstrated that Giomer restorations exhibit long-term stability, caries resistance, and excellent marginal integrity, making them an attractive alternative to conventional resin composites.^8^ Given these benefits, integrating Giomer technology into adhesive systems could enhance the performance of self-etch bonding by providing an additional protective mechanism against adhesive interface degradation.^9^

Considering these advancements, the Beautibond Xtreme adhesive system is of particular interest, as it is formulated with a unique monomer composition designed to improve bonding performance while maintaining stability over time. However, its clinical effectiveness in self-etch mode has not been fully explored. Self-etch adhesives simplify the bonding process and reduce post-operative sensitivity, but concerns remain regarding their ability to achieve long-term adhesion, especially to enamel.^10^ A clinical evaluation is essential to determine whether the self-etch application of Beautibond Xtreme can provide adequate marginal adaptation, retention, and resistance to secondary caries, particularly in Class I and II restorations, which are subjected to significant mechanical and chemical stresses.^11,12^

Thus, this randomized clinical trial aims to assess the one-year clinical performance of the Beautibond Xtreme adhesive system, comparing its performance in total-etch, self-etch, and selective enamel etching modes in Class I and II restorations. The null hypothesis of this study is that there will be no significant difference in the clinical performance of the adhesive system across the different application techniques.

## Methodology

**Figure 1:**
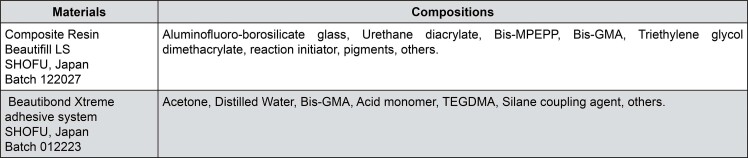
Composition of materials used in the clinical study.

This study was approved by the Ethics Committee on Human Research at the University of Ribeirão Preto (CAAE: 61246422.4.0000.5498). This clinical trial was registered at ClinicalTrials.gov under the number NCT06899386, following CONSORT 2010 guidelines. This randomized controlled clinical study evaluated a mild self-etch adhesive system, SHOFU BeautiBond Xtreme (pH≈2.5), and SHOFU Beautifil LS composite resin (both from SHOFU, Kyoto, Japan), using total-etch, selective enamel etching, and self-etching techniques in Class I and II restorations. Figure 1 shows these materials, compositions, and batch numbers.

### Study design

A total of 22 patients (14 female and 8 male), with a mean age of 45.83 years (SD = 11.24), receiving care at the Dental Clinic of the University of Ribeirão Preto (UNAERP), were selected. The study included six groups: two control groups using a universal adhesive applied in total-etch mode, and four experimental groups using self-etch and selective-etch modes. A randomized, controlled, parallel-group clinical trial was conducted, as this study design is considered the gold standard for evaluating interventions. It minimizes the influence of confounding factors and biases, thereby ensuring reliable comparisons between the bonding techniques assessed. No changes were made to the study protocol after its initiation. The study strictly followed the original plan approved by the Ethics Committee.

Patients were randomized into experimental or control groups after undergoing a clinical examination and treatment planning, according to their immediate and future needs. With the treatment plan in hand, following the priority sequence, the oral environment was prepared to begin the research. Group assignments were made by random draw for teeth that required restorative treatment and met the inclusion criteria for this study. The randomization method used a random number table generated by the Research Randomizer Program (available at: http://www.randomizer.org/form.htm). It was a simple randomization method, without stratification or block restrictions.

The sample consisted of molars and premolars that required Class I (O, OB, OP), and Class II (MO, DO, MOD) restorations, presenting active carious lesions and cavitations (radiographically indicating dentinal involvement), or unsatisfactory restorations.

Participants were given detailed information about the research and were asked to sign an informed consent form to authorize their participation. Participants could withdraw from the study at any time, for any reason, without interrupting their planned dental treatment.

For follow-up evaluations, participants were contacted one week and 24 hours prior to their appointments for confirmation, using either phone calls or a real-time messaging application (Meta Platforms, USA). Participants were informed about the importance of attending the visits and the risks of non-attendance, and were allowed a maximum of three absences. If this number was exceeded, the participant was excluded from the study. A dropout rate of approximately 20% was anticipated to ensure sufficient statistical power despite potential participant losses. While the formal sample size estimation indicated a minimum of 77 teeth, the total number was pragmatically increased to 152 to compensate for potential attrition. Participants who missed more than three scheduled follow-up visits were excluded from the final analysis. Data analysis was performed on a per-protocol basis, including only those restorations from participants who completed all evaluation time points, in line with the study’s objective to assess clinical performance under fully completed treatment and follow-up conditions.

The recruitment of participants occurred from February to April 2023. The follow-up period lasted 12 months, with the final evaluation taking place in November 2024. The study was completed as initially planned, without early termination.

All cavities were restored with Beautibond Xtreme adhesive system (Shofu Inc, Japan), and Beautifill LS composite resin (Shofu Inc, Japan). The study consisted of two control groups and four experimental groups according to the bonding techniques:

CI: Class I cavities restored with the total acid-etching technique.

CII: Class II cavities restored with the total acid-etching technique.

TI: Class I cavities restored with the self-etching technique.

TII: Class II cavities restored with the self-etching technique.

TIII: Class I cavities restored with the select-etching technique.

TIV: Class II cavities restored with the select-etching technique.

### Inclusion criteria

**Figure 2:**
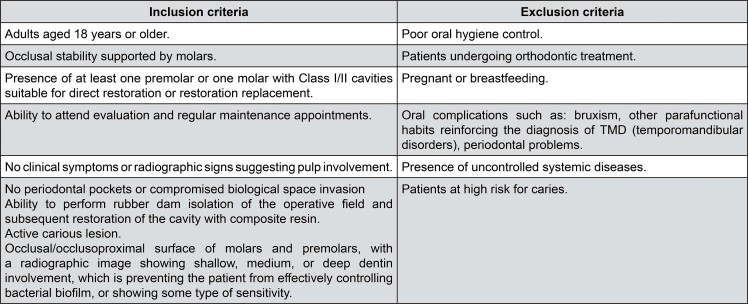
Inclusion and exclusion criteria.

Eligibility criteria included adults aged 18 years or older with good oral and systemic health, presenting at least one molar or premolar requiring a Class I or II direct restoration due to primary caries or defective restorations, and able to attend follow-up visits and undergo rubber dam isolation. Caries risk was assessed based on the American Dental Association Caries Risk Assessment guidelines, considering a combination of clinical examination and patient history. Patients were classified as high caries risk—and thus excluded—if they presented with more than one active cavitated lesion, poor oral hygiene, frequent sugar intake, or a history of high caries incidence in the previous 12 months. Additional exclusion criteria included periodontal disease, parafunctional habits, pregnancy, breastfeeding, and systemic conditions impacting oral health (Figure 2).

The restorations were performed in patients aged from 20 to 59 years. A total of 152 cavities were restored, comprising 69 Class I and 83 Class II cavities. Regarding tooth type, 93 restorations were placed in molars and 59 in premolars. Of these, 82 teeth were located in the upper jaw and 70 in the lower jaw. The majority of the dentin involved in the restorations was affected by caries without a cavitated base.

### Operative technique

All restorative procedures were performed by a single experienced operator who underwent a calibration process prior to patient recruitment. The calibration consisted of practical training that included restoring ten posterior teeth in phantom heads and five clinical cases fulfilling the same inclusion criteria as the trial. These procedures were conducted under the supervision of two senior specialists in restorative dentistry. The calibration aimed to ensure strict adherence to standardized protocols for cavity preparation, adhesive application (total-etch, self-etch, or selective enamel etching), composite resin placement, and light curing, in accordance with the manufacturer’s instructions. Full agreement between the operator and the supervisors regarding each procedural step was achieved before initiating the clinical phase. Rubber dam isolation was achieved using a rubber dam after prophylaxis with pumice and water, and local anesthesia was administered when necessary. The operator was not blinded to the bonding technique used.

Participants were not informed about the adhesive protocol (total-etch, self-etch, or selective enamel etching) assigned to their restorations. This approach was feasible because all clinical procedures, from the patient’s perspective, followed the same general workflow involving cavity preparation, adhesive application, composite restoration, and finishing. The differences between adhesive protocols are subtle and occur primarily in operative steps that are indistinguishable to the patient, such as whether acid etching is applied to enamel or dentin.

### Cavity preparation

Cavity dimensions were determined by the removal of carious tissue and/or unsatisfactory restorations, guided by visual (clinical and radiographic) and tactile inspection. Carious tissue was removed using spherical carbide burs of a diameter compatible with the cavity, at low speed, and assisted by dentin excavators. Carious tissue was completely removed from the surrounding walls and floor until the remaining dental tissue resisted cutting with a manual instrument. In cases of deep carious lesions posing a risk of pulp exposure, carious tissue was completely removed from the surrounding walls, with partial removal on the floor to avoid pulp exposure.^10^ The pulp tissue’s response capacity was assessed before proceeding with the restorative treatment. If replacement of an old, defective restoration was necessary, or if access to carious tissue through the enamel was required, the procedure was conducted with a spherical diamond tip at high speed, under water/air cooling. The cavosurface angle was sharp, without a bevel. After completing preparation and removal of carious tissue, the cavity was rinsed with a water/air spray.

### Restorative procedure

All restorative procedures were performed by an experienced dentist following the manufacturer’s recommended protocol. The groups were restored as follows:

Group CI and CII - BeautiBond Xtreme (total-etch technique) + Beautifil LS: Enamel was etched for 30 seconds and dentin for 15 seconds using 35% phosphoric acid gel (Ultradent). The acid was then rinsed, and excess moisture was removed with an air spray. To protect the dentin during air blasting, an absorbent paper was held over it with clinical tweezers, ensuring a whitish appearance for the enamel and slightly moist dentin. Excess water on the cotton roll was also dried. The BeautiBond Xtreme adhesive system was applied without waiting, followed by a gentle air spray for 3 seconds and then a strong air blast to ensure solvent evaporation. Finally, the adhesive was light-cured for 5 seconds using a LED photopolymerizer (Gran Valo LED Curing Light, Ultradent) at a light intensity of 1600 mW/cm^2^.

Group TI and TII – BeautiBond Xtreme (self-etch technique) + Beautifil LS: No acid etching was performed on either the enamel or dentin. The BeautiBond Xtreme adhesive system was applied immediately, without waiting, followed by a gentle air spray for 3 seconds and then a strong air blast to ensure solvent evaporation. The adhesive was then light-cured for 5 seconds at a light intensity of 1600 mW/cm^2^.

Group TIII and TIV – BeautiBond Xtreme (selective acid-etch technique) + Beautifil LS: Enamel was etched for 30 seconds using 35% phosphoric acid gel (Ultradent). The acid was then rinsed, and excess moisture was removed with an air spray, leaving the enamel with a whitish appearance. Excess water on the cotton roll was also dried. Next, the BeautiBond Xtreme adhesive system was applied immediately, without waiting, followed by a gentle air spray for 3 seconds and then a strong air blast to ensure solvent evaporation. The adhesive was then light-cured for 5 seconds at a light intensity of 1600 mW/cm^2^.

Beautifil LS composite resin was inserted using the stratified technique, with each increment not exceeding 2 mm. The resin was placed horizontally, bonding all walls due to its low polymerization shrinkage of 0.85% by volume. Each increment was then light-cured for 20 seconds at a light intensity of 1000 mW/cm^2^.

### Clinical and radiographic evaluation

All patients participating in this study received guidance on the etiology, prevention, and control of dental caries and periodontal disease, and participated in a program of periodic follow-up and professional maintenance. The initial evaluation was performed one week after the restoration, during which the finishing and polishing of the restoration were carried out, along with the assessment of any possible clinical symptoms related to post-operative sensitivity or pain.

Patients returned one month after polishing for further evaluation of the previously described criteria, and followed up every 6 months for additional assessments and radiographic monitoring.

For clinical evaluations, photographs were taken by a trained operator after prophylaxis, with the operative field dry and using an intraoral photographic mirror. A Canon Rebel G EOS camera with a 100mm macro lens and a 1:1 adapter was used for capturing high detail small images. No external light was used, only a circular flash for macro lenses.

For the radiographic evaluation, a film holder and a Spectro 70X X-ray machine (Dabi Atlante, Ribeirão Preto, SP, Brazil) with 70 kVp, 8 mA, and an exposure time of 0.3 seconds were used. The sensitized images, after radiographic capture, were introduced into the laser optical reader of the Digora digital radiographic system (Soredex Orion Corporation, Helsinki, Finland), which processed the image. The patient was always protected with a lead apron. All radiographic images were evaluated using Scanora software to observe possible bone rarefactions, periodontal ligament thickness, signs of marginal restoration adaptation failure, suggestive signs of caries and/or infiltration, and restorative adaptation failure.

Clinical and radiographic evaluations were conducted at 7 days, 30 days, and 6 months, with a total follow-up period of 1 year. To avoid assessment bias, the clinical operator did not participate in the postoperative evaluations. The two examiners responsible for the clinical evaluations were experienced dentists who underwent a calibration process prior to the beginning of the study. The training involved the assessment of 20 posterior restorations in patients who were not part of the main trial, using the same modified USPHS criteria adopted for the study. This process was supervised by a senior researcher with expertise in restorative dentistry to ensure the correct and consistent application of all evaluation parameters.

**Figure 3:**
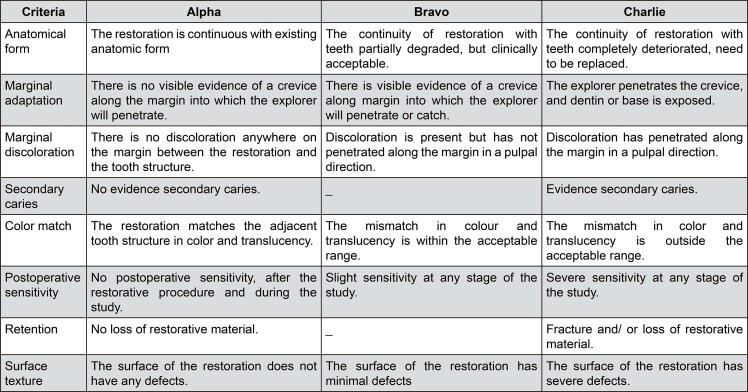
Modified United States Public Health Service (USPHS) criteria used in this study.

To assess reliability, both inter- and intra-examiner agreements were tested. Each examiner repeated the evaluation of the same set of 20 restorations after a two-week interval. The inter-examiner kappa coefficient was 0.84, indicating excellent agreement. Intra-examiner reliability showed kappa values of 0.81 for examiner 1 and 0.88 for examiner 2, confirming high consistency in the evaluations. The examiners were blinded to the restorative protocol used. All 14 restorations were inspected and evaluated according to the codes and criteria based on a modification of those established by Ryge and Cvar (Figure 3).^13,14^

Although ‘anatomical form’ is more closely associated with the mechanical properties and wear resistance of the composite resin, it was included in the evaluation in accordance with the USPHS criteria to allow comprehensive and standardized assessment.

### Sample size calculation

The sample size was estimated using G*Power 3.1 software, considering *marginal adaptation* as the primary outcome. This parameter was selected because it is widely recognized as a sensitive and clinically relevant indicator of adhesive performance, particularly in relation to the long-term stability of the tooth-restoration interface. Previous studies have shown that marginal adaptation is one of the earliest parameters to exhibit deterioration when bonding protocols are inadequate.

The estimation was based on an expected effect size of 0.6, a significance level (α) of 0.05, and a statistical power (1-β) of 95%. The analysis indicated that a minimum of 77 restorations would be required to detect statistically significant differences between groups. To compensate for potential dropouts and ensure adequate representation, the final sample size was increased to 152 restorations.

### Clinical outcomes and statistical analysis

In this study, the primary outcomes were marginal adaptation, marginal discoloration, secondary caries, postoperative sensitivity, and retention, as these criteria are directly related to the clinical performance and durability of adhesive restorations. The secondary outcomes included anatomical form, color match, and surface texture, which reflect esthetic and surface-level characteristics of the restorations. All outcomes were assessed according to the modified USPHS criteria at baseline, 6 months, and 12 months.

The collected data were summarized in Table 1 and analyzed using non-parametric methods appropriate for ordinal data. The Kruskal-Wallis test was used to compare outcomes between groups at each time point, whereas the Friedman test was employed to assess changes within groups over time. The significance level was set at 5% for all analyses.

**Table 1:** Absolute and relative frequency (percentage) of the evaluation of restorations according to modified criteria (Alpha, Bravo, Charlie) at baseline, 6 months, and 1 year for the different experimental groups.

CRITERIA	BASELINE			6-MONTH			ONE-YEAR		
	A	B	C	A	B	C	A	B	C
**Anatomical Form**									
CI	26 (100)^Aa^	0	0	26 (100)^Aa^	0	0	26 (92.9)^Aa^	0	0
CII	30 (100)^Aa^	0	0	30 (100)^Aa^	0	0	30 (100)^Aa^	0	0
TI	19 (100)^Aa^	0	0	17 (89.47)^Aa^	2 (10.53)	0	17 (89.47)^Aa^	2 (10.53)	0
TII	28 (100)^Aa^	0	0	26 (92.86)^Aa^	2 (7.14)	0	26 (92.86)^Aa^	2 (7.14)	0
TIII	22 (100)^Aa^	0	0	22 (100)^Aa^	0	0	22 (95.6)^Aa^	0	0
TIV	27 (100)^Aa^	0	0	27 (100)^Aa^	0	0	27 (100)^Aa^	0	0
**Marginal adaptation**									
CI	26 (100)^Aa^	0	0	26 (100)^Aa^	0	0	26 (100)^Aa^	0	0
CII	30 (100)^Aa^	0	0	29 (96.7)^Aa^	1 (3.3)	0	29 (96.7)^Aa^	1 (3,3)	0
TI	19 (100)^Aa^	0	0	13 (68.42)^Bb^	6 (31.58)	0	13 (68.42)^Bb^	6 (31.58)	0
TII	28 (100)^Aa^	0	0	19 (67.86)^Bb^	9 (32.14)	0	19 (67,86)^Bb^	9 (32.14)	0
TIII	22 (100)^Aa^	0	0	21 (95.5)^Aa^	1 (4.5)	0	21 (95.5)^Aa^	1 (4.5)	0
TIV	27 (100)^Aa^	0	0	26 (96.3)^Aa^	1 (3.7)	0	26 (96.3)^Aa^	1 (3.7)	0
**Marginal discoloration**									
CI	26 (100)^Aa^	0	0	26 (100)^Aa^	0	0	26 (100)^Aa^	0	0
CII	30 (100)^Aa^	0	0	30 (100)^Aa^	0	0	30 (100)^Aa^	0	0
TI	19 (100)^Aa^	0	0	13 (68.42)^Bb^	6 (31.58)	0	13 (68.42)^Bb^	6 (31.58)	0
TII	28(100)^Aa^	0	0	19 (67.86)^Bb^	9 (32.14)	0	19 (67.86)^Bb^	9 (32.14)	0
TIII	22 (100)^Aa^	0	0	22 (100)^Aa^	0	0	22 (100)^Aa^	0	0
TIV	27 (100)^Aa^	0	0	27 (100)^Aa^	0	0	27 (100)^Aa^	0	0
**Secondary caries**									
CI	26 (100)^Aa^	0	0	26 (100)^Aa^	0	0	26 (100)^Aa^	0	0
CII	30 (100)^Aa^	0	0	30 (100)^Aa^	0	0	30 (100)^Aa^	0	0
TI	19 (100)^Aa^	0	0	19 (100)^Aa^	0	0	19 (100)^Aa^	0	0
TII	28 (100)^Aa^	0	0	28 (100)^Aa^	0	0	28 (100)^Aa^	0	0
TIII	22 (100)^Aa^	0	0	22 (100)^Aa^	0	0	22 (100)^Aa^	0	0
TIV	27(100)^Aa^	0	0	27 (100)^Aa^	0	0	27 (100)^Aa^	0	0
**Color match**									
CI	26(100)^Aa^	0	0	25 (96.1)^Aa^	1 (3,9)	0	25 (96.1)	1 (3.9)	0
CII	30 (100)^Aa^	0	0	30 (100)^Aa^	0	0	30 (100)	0	0
TI	19 (100)^Aa^	0	0	19 (100)^Aa^	0	0	19 (100)	0	0
TII	28 (100)^Aa^	0	0	28 (100)^Aa^	0	0	28 (100)	0	0
TIII	22(100)^Aa^	0	0	18 (81.8)^Ab^	4 (18,2)	0	18 (81.8)^Ab^	4 (18.2)	0
TIV	27 (100)^Aa^	0	0	26 (96.3)^Aa^	1 (3,7)	0	26 (96.3)^Aa^	1 (3.7)	0
**Postoperative sensitivity**									
CI	26 (100)^Aa^	0	0	26 (100)^Aa^	0	0	26 (100)^Aa^	0	0
CII	30(100)^Aa^	0	0	30 (100)^Aa^	0	0	30 (100)^Aa^	0	0
TI	19(100)^Aa^	0	0	19 (100)^Aa^	0	0	19 (100)^Aa^	0	0
TII	28(100)^Aa^	0	0	28 (100)^Aa^	0	0	28 (100)^Aa^	0	0
TIII	22 (100)^Aa^	0	0	22 (100)^Aa^	0	0	22 (100)^Aa^	0	0
TIV	27 (100)^Aa^	0	0	27 (100)^Aa^	0	0	27 (100)^Aa^	0	0
**Retention**									
CI	26 (100)^Aa^	0	0	26 (100)^Aa^	0	0	26 (100)^Aa^	0	0
CII	30 (100)^Aa^	0	0	30 (100)^Aa^	0	0	30 (100)^Aa^	0	0
TI	19 (100)^Aa^	0	0	19 (100)^Aa^	0	0	19 (100)^Aa^	0	0
TII	28 (100)^Aa^	0	0	28 (100)^Aa^	0	0	28 (100)^Aa^	0	0
TIII	22 (100)^Aa^	0	0	22 (100)^Aa^	0	0	22 (100)^Aa^	0	0
TIV	27 (100)^Aa^	0	0	27 (100)^Aa^	0	0	27 (100)^Aa^	0	0
**Surface texture**									
CI	26 (100)^Aa^	0	0	26 (100)^Aa^	0	0	26 (100)^Aa^	0	0
CII	30 (100)^Aa^	0	0	30 (100)^Aa^	0	0	30 (100)^Aa^	0	0
TI	19 (100)^Aa^	0	0	19 (100)^Aa^	0	0	19 (100)^Aa^	0	0
TII	28 (100)^Aa^	0	0	28 (100)^Aa^	0	0	28 (100)^Aa^	0	0
TIII	22 (100)^Aa^	0	0	22 (100)^Aa^	0	0	22 (100)^Aa^	0	0
TIV	27 (100)^Aa^	0	0	27 (100)^Aa^	0	0	27 (100)^Aa^	0	0

*Uppercase letters denote differences among groups at a single time point (Kruskal-Wallis test p<0.05).

Lowercase letters indicate differences within the same group over time (Friedman test p<0.05).

## Results

The evaluation of Class I and II restorations using the BeautiBond Xtreme adhesive system under different etching protocols was conducted over a one-year period. Clinical assessments were performed at baseline, six months, and one year, using the modified United States Public Health Service (USPHS) criteria. All evaluations were conducted by two calibrated, blinded examiners (inter-examiner kappa coefficient was 0.84; intra-examiner kappa values of 0.81 for examiner 1 and 0.88 for examiner 2) to ensure consistency. Additionally, no changes were made to the predefined primary and secondary outcomes after the study began. All outcomes were assessed as originally planned.

A total of 155 restorations were placed in patients aged over 18 years. Of these, 152 restorations were evaluated at both 6-month and one-year recalls, representing a recall rate of 98.06%. Three restorations (from one patient) were lost due to participant withdrawal. Of these, 65 restorations (42.8%) were placed in premolars, and 87 (57.2%) were placed in molars. Clinical evaluation scores at baseline, six months, and one year were analyzed and showed no significant differences between premolars and molars for all parameters and evaluation periods (p>0.05).

After one year, survival rates of all groups were 100%.

Retention was consistent across all groups (CI, CII, TI, TII, TIII, TIV), with no loss of restorative material observed at baseline, six months, or one year. All restorations received an “Alpha” score, indicating complete retention throughout the evaluation period (p>0.05).

The anatomical form was well-preserved across all restorations at baseline, with 100% receiving an “Alpha” score. At six months and one year, no significant deviations were observed across all groups. However, slight degradation in anatomical form was noted in groups TI and TII, with 10.53% and 7.14% of restorations, respectively, receiving “Bravo” scores at both the six-month and one-year follow-ups. Despite this observation, the differences were not statistically significant (p>0.05).

All groups exhibited ideal marginal adaptation at baseline (p>0.05). At six months and one year, significantly more Bravo scores were observed for self-etch Class I and II restorations (TI and TII, respectively) compared to those in the total-etch (CI and CII) and selective enamel etching groups (TIII and TIV). At six months, pairwise comparisons revealed significant differences for TI vs. CI (p=0.0021), TI vs. CII (p=0.049), TI vs. TIII (p=0.024), TI vs. TIV (p=0.035), TII vs. CI (p=0.0018), TII vs. CII (p=0.045), TII vs. TIII (p=0.049), and TII vs. TIV (p=0.034). At one year, the same pattern was observed with even stronger significance: TI vs. CI (p=0.00001), TI vs. CII (p=0.0007), TI vs. TIII (p=0.0039), TI vs. TIV (p=0.0011), TII vs. CI (p=0.00009), TII vs. CII (p=0.01), TII vs. TIII (p=0.024), and TII vs. TIV (p=0.01).

No discoloration was observed at baseline in any of total-etch or selective enamel etching groups (p>0.05). However, by six months, significant minor discoloration was observed in the self-etch groups, with 27.78% and 32.14% of restorations in TI and TII, respectively, receiving “Bravo” scores. Pairwise comparisons confirmed statistically significant differences compared to the total-etch and selective enamel etching groups, with p = 0.0036 for TI and p = 0.0095 for TII. These results remained consistent at the one-year follow-up, with no additional changes observed (p>0.05).

Baseline evaluations showed excellent color match in all groups, with 100% receiving “Alpha” scores. At six months and one year, slight mismatches were recorded in groups CI and TIV, in which 3.9% and 3.7% of restorations, respectively, received “Bravo” scores (p=0.0010 for CI and p=0.0011 for TIV). Group TIII experienced greater color match degradation, with 18.2% receiving “Bravo” by the one-year evaluation (p<0.00001).

Surface texture evaluations revealed no defects at baseline across all groups. This remained unchanged at 6 months and one year, with all restorations retaining “Alpha” scores.

No evidence of secondary caries was observed in any group at baseline, 6 months, or one year. All restorations received “Alpha” scores throughout the evaluation period.

No postoperative sensitivity was reported at baseline, 6 months, or one year across all groups. Each restoration consistently received an “Alpha” score (p>0.05).

No adverse effects or undesirable events were reported during the one-year follow-up period in any of the evaluated groups.

**Figure 4:**
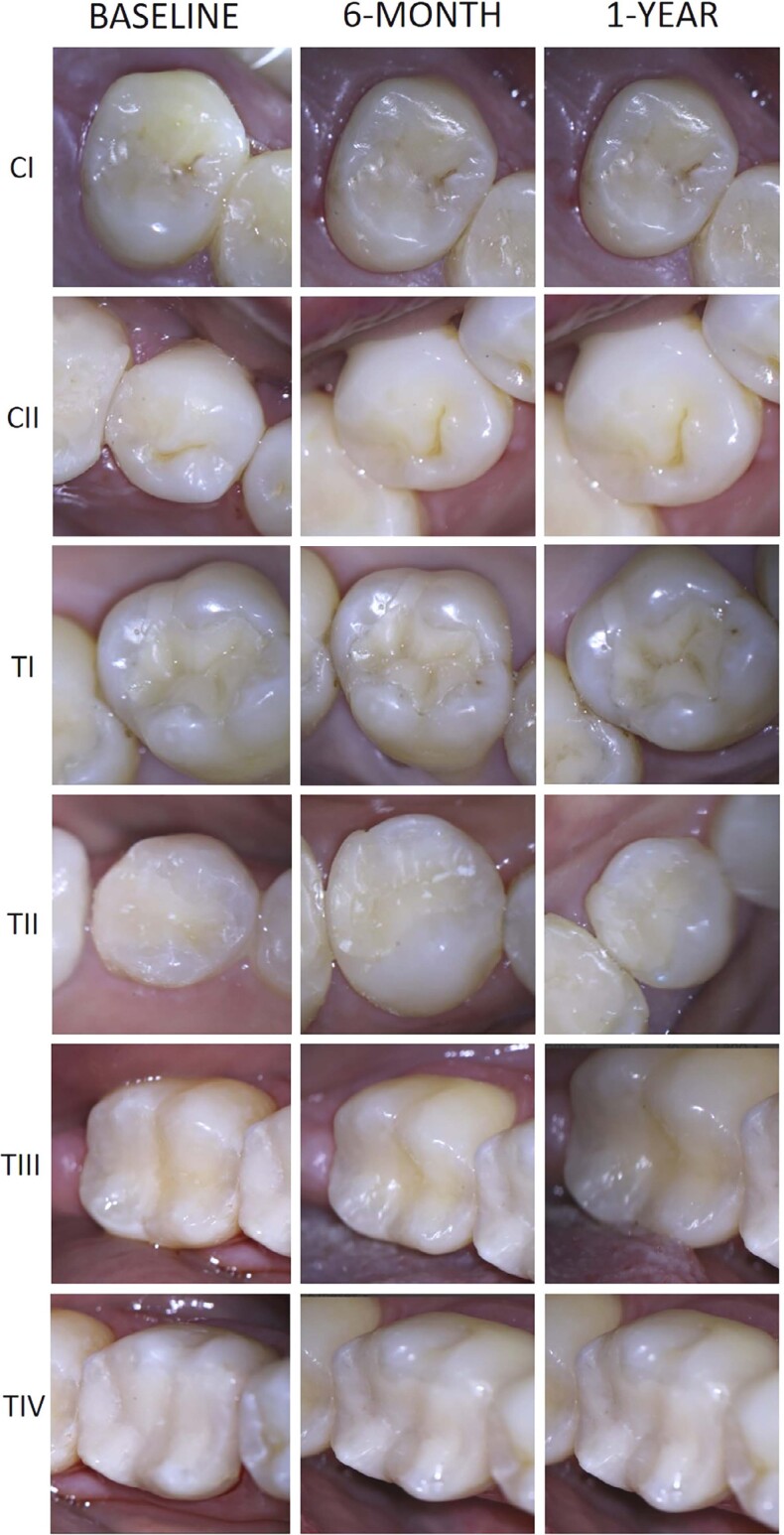
Clinical photographs of the restorations for all experimental groups at baseline, 6 months, and 1 year.

**Figure 5:**
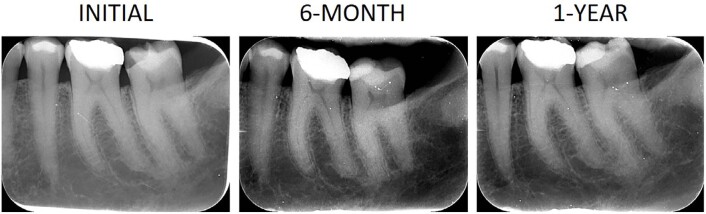
Representative radiographic follow-up of the study, performed at the initial time point (pre-restoration), 6 months, and 1 year after the restoration.

Representative images below show clinical photographs of the restorations from the different experimental groups at baseline, 6 months, and 1 year (Figure 4), and a representative radiographic follow-up, performed at baseline, 6 months, and 1 year after restoration (Figure 5).

## Discussion

This clinical study aimed to assess the performance of the BeautiBond Xtreme adhesive system using total-etch, self-etch, and selective enamel etching techniques in Class I and Class II restorations. The evaluation criteria included anatomical form, marginal adaptation, marginal discoloration, color match, surface texture, secondary caries, postoperative sensitivity, and retention. The findings indicated that the total-etch and selective enamel etching approaches outperformed the self-etch technique, thus rejecting the null hypothesis.

The continuous development of new restorative materials and techniques has led to a scarcity of long-term clinical assessments. While laboratory studies play an essential role in the initial evaluation and development of restorative materials, they attempt to simulate clinical conditions but cannot fully replicate the complexities of the oral environment. Due to the multiple variables present in the mouth, their findings may not precisely predict *in vivo* performance.^15^ Consequently, carefully designed randomized controlled clinical trials are necessary to accurately assess the clinical behavior of new materials and to compare the effectiveness of different restorative options.^16^

A major challenge in ensuring the durability of dental restorations is controlling multiple factors, such as the adhesive properties of the restorative material, its application and polymerization technique, as well as the dimensions and design of the restoration. Additionally, the clinician’s expertise in material handling and patient-specific factors, such as occlusal forces, fluctuations in intraoral temperature, and pH variations, also play a significant role in the longevity of restorations.^15,17^

The adhesive system used in this study, BeautiBond Xtreme, was combined with Beautifil LS composite resin to standardize the materials across all restorations. Although the study included both Class I and Class II cavity preparations, the groups were allocated using a simple randomization method without stratification by cavity class. Consequently, some imbalance occurred in the baseline distribution of teeth across the experimental groups (e.g., one group included 26 teeth, while another had 19). While the randomized design allowed for unbiased allocation, the lack of stratification may have influenced the comparability of groups in terms of initial cavity characteristics. This imbalance represents a limitation, particularly given the per-protocol analysis approach used in this study. Despite consistent application of adhesive protocols regardless of cavity class and no significant differences observed between Class I and II restorations for any clinical outcome, the uneven group sizes should be considered when interpreting the results. Additionally, to reduce operator-related variability, all restorative procedures were performed by a single clinician with advanced training in operative dentistry. While this enhanced the standardization of the technique, it may also limit the external validity of the findings, as clinical outcomes can vary with operator skill and experience.

To ensure an objective and reliable assessment of restorative materials in clinical trials, standardized evaluation criteria are essential. The modified USPHS criteria were selected for the clinical evaluation in this study because they represent one of the most widely adopted and validated systems in adhesive dentistry research. This method has been extensively used in long-term clinical trials assessing the performance of direct composite restorations, particularly in posterior teeth.^18^ Its widespread application enables direct comparison with a substantial body of existing literature and provides continuity in the interpretation of clinical outcomes across studies.^7^ Moreover, the USPHS criteria offer sufficient sensitivity to detect clinically relevant changes in key parameters, such as marginal adaptation, marginal discoloration, anatomical form, surface texture, postoperative sensitivity, secondary caries, and retention. Another important advantage is the high reproducibility between examiners, which contributes to the reliability of the longitudinal assessments performed in this trial. Therefore, the adoption of USPHS criteria was considered appropriate to meet the objectives of this study, which focused on comparing the clinical behavior of different adhesive protocols over time.^15,18^ All restorations were assessed under these criteria by two calibrated and experienced examiners, ensuring consistency and accuracy in the clinical performance analysis of the tested adhesive system and composite resin.^19^

Giomer technology represents an innovative class of restorative materials that combines the benefits of resin composites with bioactive properties derived from glass ionomers. These materials are based on Surface Pre-Reacted Glass-ionomer (S-PRG) technology, in which fluoroboroaluminosilicate glass particles undergo a controlled acid-base reaction before being incorporated into a resin matrix.^8^ This structure enables the continuous release and recharge of multiple ions, including fluoride, which contribute to acid neutralization, inhibition of bacterial activity, and promotion of enamel remineralization. These bioactive effects may play an important role in reducing the risk of secondary caries.^20^ Compared to conventional composite resins, giomers maintain the esthetic and mechanical advantages of resin-based materials while offering additional protective benefits due to the sustained release of fluoride and other bioactive ions.^8^ Furthermore, studies have demonstrated that the fluoride release in giomers is more stable over time than that of other fluoride-releasing restorative materials, making them a promising choice for long-term caries prevention.^8,20^ Corroborating previous findings, this study observed no secondary caries lesions, regardless of the adhesion mode or cavity type. However, while the ion-releasing capability of giomer-based materials may contribute to a protective effect, it should be emphasized that caries prevention is multifactorial and highly dependent on patient-specific factors such as biofilm control and dietary habits.

Clinical studies with extended follow-up periods have demonstrated that giomer-based restorations perform comparably to conventional resin composites regarding key parameters such as retention, marginal adaptation, and surface quality.^7,8^ A five-year study evaluating Beautifil II and Beautifil Flow Plus F00, both nano-hybrid giomer composites with different viscosities, found that these materials maintained stable clinical performance over time, showing no significant differences in color stability, marginal discoloration, or secondary caries incidence compared to traditional resin composites.^8^ Despite differences in formulation, specifically in filler load, viscosity, and application technique, these long-term results reinforce the potential durability of giomer-based restorative materials. In the present study, after one year of follow-up, no clinically relevant changes were observed for any bonding protocol for anatomic form, color match, surface texture, or retention. These short-term findings are consistent with previous long-term studies and support the clinical viability of the adhesive system and restorative procedures evaluated.

The etch-and-rinse mode involves applying phosphoric acid to completely remove the smear layer, creating a porous dentin network where adhesive monomers infiltrate, forming a robust hybrid layer through micromechanical retention. In contrast, the self-etch mode uses acidic monomers to modify the smear layer rather than fully removing it, leading to a milder demineralization of dentin while preserving some smear plugs within dentinal tubules, which aids reduce nanoleakage and enhances bond durability.^10,21-23^ The selective enamel etching technique combines these approaches by etching only the enamel surface with phosphoric acid while maintaining the self-etch approach on dentin, optimizing adhesion to both substrates. This versatility makes universal adhesives practical for various clinical situations, allowing dentists to choose the most suitable bonding strategy based on the cavity configuration and substrate characteristics.^24-26^

Studies comparing the bonding effectiveness of universal adhesives across different etching techniques indicate that the choice of conditioning mode influences adhesion durability, particularly in enamel. *In vitro* studies suggest that, for dentin, self-etching can achieve similar immediate and long-term bond strength as the etch-and-rinse technique, particularly when using mild universal adhesives.^23,24^ However, when bonding to enamel, selective etching with phosphoric acid improves bond strength, as the self-etch approach alone does not create sufficient micromechanical retention.^24^

From a clinical perspective, there is evidence that restorations placed with self-etch adhesives exhibit stable retention and marginal adaptation over time.^10,23-25^ However, selective enamel etching has been associated with improved marginal integrity, reducing the likelihood of marginal staining and restoration failure.^24^ These findings suggest that the combination of self-etching on dentin and selective etching on enamel is an effective strategy for optimizing both bond stability and clinical performance, aligning with current adhesive dentistry recommendations. Supporting previous evidence,^10,23-25^ this study observed that, at both six-month and one-year follow-ups, teeth restored with the self-etch adhesive system exhibiter lower marginal integrity and more pronounced staining compared to baseline.

The findings of this study reinforce the clinical relevance of selecting appropriate adhesive protocols. Notably, the selective enamel etching strategy demonstrated favorable clinical outcomes, supporting its use as a viable approach to optimize enamel bonding without compromising dentin application. In particular, adhesives formulated with 10-MDP and S-PRG fillers have shown favorable bonding performance and reduced interfacial degradation *in vitro*, supporting the potential of Beautibond Xtreme for long-term clinical success. For instance, Cuevas-Suárez, et al. (2019)^25^ demonstrated that universal adhesives containing 10-MDP exhibited higher bond strength and more durable performance compared to those without functional monomers. Additionally, the comprehensive review by Breschi (2025)^10^ highlighted the advantages of selective enamel etching when using self-etch adhesives, especially those designed for reduced technique sensitivity and enhanced chemical bonding. These results are further supported by recent systematic reviews on universal adhesives, which emphasize the role of functional monomers and application protocols in achieving stable adhesion over time.^10,23,24^

Despite the observed marginal integrity compromise in some restorations, no secondary caries lesions were detected during the follow-up period. While this outcome may be partially associated with the ion-releasing properties of the bioactive materials used—such as Beautibond Xtreme adhesive and Beautifil LS Giomer composite, both containing surface pre-reacted glass-ionomer (S-PRG) fillers—a cautious interpretation is warranted. These materials are known to release fluoride, strontium, and borate ions, which can enhance enamel and dentin remineralization and exhibit antimicrobial effects, potentially contributing to a more stable and protective interface. Fluoride ions promote the formation of fluorapatite, increasing resistance to acid attacks, while borate and strontium ions have been shown to inhibit bacterial adhesion and metabolic activity, thereby reducing the risk of recurrent caries.^20^ In addition, previous studies have demonstrated that giomer-based restorations maintain their ion-releasing capacity over extended periods, thus supporting a long-term protective effect against demineralization and bacterial colonization.^8,20^ However, it is important to consider that all participants in the present study were classified as low caries risk individuals. Therefore, the absence of secondary lesions cannot be attributed solely to the restorative materials. Dental caries is a multifactorial disease, and patient-related factors such as oral hygiene habits, dietary patterns, and salivary flow also play critical roles in its prevention. In this context, the favorable clinical outcomes observed are likely the result of a synergistic interaction between the bioactive properties of the restorative materials and the individual characteristics of the patient population. This interpretation is consistent with current evidence on caries risk assessment and supports a comprehensive, multifactorial perspective when evaluating restorative performance.

The clinical findings of the present study are consistent with recent laboratory evidence demonstrating that the adhesive performance of self-etch systems can be significantly influenced by their chemical composition, particularly the presence and interaction of functional monomers, bioactive particles, and HEMA. Costa, et al. (2024)^9^ reported that self-etch adhesives applied to simulated altered dentin exhibited reduced bond strength and interfacial stability when formulations contained suboptimal concentrations or ineffective combinations of functional monomers. Moreover, the study highlighted that the presence of bioactive particles alone was insufficient to compensate for the lower etching capacity of mild self-etch adhesives, particularly in substrates such as enamel or, hypothetically, sclerotic dentin, where the mineralized surface could reduce micromechanical interlocking.

The 12-month clinical performance of BeautiBond Xtreme observed in this study, particularly in terms of marginal adaptation and discoloration, can be compared to other mild universal adhesives with similar pH profiles. For example, studies evaluating adhesives such as Clearfil SE Bond and Scotchbond Universal, which also exhibit pH values around 2.5, have reported comparable outcomes in short-term follow-ups, especially when applied using selective enamel etching protocols. These parallels suggest that, despite its mild acidity, BeautiBond Xtreme demonstrates clinical effectiveness when combined with appropriate bonding strategies.^23-25^

In this context, the inferior clinical performance observed in the self-etch groups of the present study, particularly regarding marginal adaptation and marginal discoloration, may be partly attributed to the inherent chemical limitations of the adhesive formulation when applied without prior enamel etching. This reinforces the importance of adopting bonding strategies that optimize micromechanical retention, such as selective enamel etching or total-etch approaches, especially in restorations subject to significant mechanical and chemical stresses, such as those in posterior teeth. Despite the lack of statistical differences between cavity types in this study, the biomechanical complexity of proximal contacts and marginal ridges in Class II preparations is a well-documented risk factor for marginal breakdown over time.^27,28^ This highlights the importance of bonding strategies that optimize both micromechanical and chemical adhesion, especially in stress-bearing areas.

A limitation of this study is the relatively short follow-up period. While long-term assessments are essential for a more comprehensive understanding of the clinical behavior of restorative materials, short-term evaluations also provide valuable insights into their performance in real clinical conditions. In this study, changes in marginal staining and integrity were already observed at six months and remained stable up to one year. Despite the limited timeframe of this evaluation, patient follow-up will continue to allow further analysis of the materials’ long-term effectiveness.

Another limitation of this study is the absence of a split-mouth design, which is often used to minimize inter-patient variability, such as differences in oral hygiene, diet, and brushing habits, which can all influence the longevity of restorations. However, while split-mouth studies enhance internal validity, they also pose challenges, including a higher risk of patient dropout, as a missed follow-up appointment could result in the loss of multiple restorations from the analysis. In this study, a parallel-group randomized design was adopted, ensuring that all included patients met strict selection criteria, which helped to control confounding variables. Additionally, the methodology employed—such as standardized restorative procedures, calibrated evaluators (inter-examiner kappa coefficient was 0.84, while the intra-examiner kappa values were 0.81 for examiner 1 and 0.88 for examiner 2), and strict follow-up protocols—supports the reliability of the findings, reinforcing the consistency of the clinical outcomes despite variations in individual patient habits.

Another methodological consideration is that the study employed a per-protocol analysis, which included only those restorations that completed the full follow-up period according to the study protocol. While this approach enhances internal validity and ensures the results reflect the outcomes of fully implemented adhesive procedures, it may also limit the generalizability of the findings. Patients who fail to adhere to follow-up schedules or require retreatment are excluded from the analysis, potentially leading to an overestimation of treatment effectiveness under ideal conditions. Therefore, although the clinical outcomes reported herein are promising, they should be interpreted with caution and viewed in the context of a controlled clinical setting with strict protocol adherence.

The findings of this randomized clinical trial reinforce the critical role of adhesive strategy selection in determining the clinical performance of posterior composite restorations. The total-etch and selective enamel etching techniques demonstrated superior marginal integrity and reduced marginal staining compared to the self-etch approach, highlighting the importance of optimizing enamel bonding through selective phosphoric acid etching. Moreover, the absence of secondary caries and restoration failures over the 12-month follow-up period supports the clinical effectiveness of the adhesive protocol when combined with a giomer-based restorative material. Despite the limitations related to the follow-up duration, single-operator execution, and parallel-group design, the study provides relevant insights that align with existing evidence and contribute to the body of knowledge on universal adhesives. Continued patient follow-up is underway to assess the long-term durability of the tested protocols. Future studies with extended follow-up periods, multicenter designs, and larger sample sizes are encouraged to validate and expand upon these findings, enhancing their generalizability to diverse clinical settings.

## Conclusion

After one year of clinical evaluation, the BeautiBond Xtreme adhesive system demonstrated superior performance when applied with total-etch and selective enamel etching protocols, with better marginal integrity and less marginal staining compared to the self-etch approach. No cases of secondary caries or restoration loss were observed during this period, supporting the clinical effectiveness of the adhesive procedures evaluated within the limitations of a 12-month follow-up.
